# Utility of neutrophil-to-lymphocyte ratio as an indicator of tumor immune status in non-small cell lung cancer

**DOI:** 10.1093/jjco/hyae058

**Published:** 2024-05-04

**Authors:** Kazuma Iwata, Ken Suzawa, Kohei Hashimoto, Shin Tanaka, Kazuhiko Shien, Kentaroh Miyoshi, Hiromasa Yamamoto, Mikio Okazaki, Seiichiro Sugimoto, Shinichi Toyooka

**Affiliations:** Department of General Thoracic Surgery and Brest and Endocrinological Surgery, Okayama University Graduate School of Medicine, Dentistry and Pharmaceutical Sciences, 2-5-1 Shikata-cho, Kita-ku, Okayama 700-8558, Japan; Department of General Thoracic Surgery and Brest and Endocrinological Surgery, Okayama University Graduate School of Medicine, Dentistry and Pharmaceutical Sciences, 2-5-1 Shikata-cho, Kita-ku, Okayama 700-8558, Japan; Department of Thoracic Surgery, Okayama University Hospital, 2-5-1 Shikata-cho, Kita-ku, Okayama 700-8558, Japan; Department of General Thoracic Surgery and Brest and Endocrinological Surgery, Okayama University Graduate School of Medicine, Dentistry and Pharmaceutical Sciences, 2-5-1 Shikata-cho, Kita-ku, Okayama 700-8558, Japan; Department of Thoracic Surgery, Okayama University Hospital, 2-5-1 Shikata-cho, Kita-ku, Okayama 700-8558, Japan; Department of General Thoracic Surgery and Brest and Endocrinological Surgery, Okayama University Graduate School of Medicine, Dentistry and Pharmaceutical Sciences, 2-5-1 Shikata-cho, Kita-ku, Okayama 700-8558, Japan; Department of Thoracic Surgery, Okayama University Hospital, 2-5-1 Shikata-cho, Kita-ku, Okayama 700-8558, Japan; Department of General Thoracic Surgery and Brest and Endocrinological Surgery, Okayama University Graduate School of Medicine, Dentistry and Pharmaceutical Sciences, 2-5-1 Shikata-cho, Kita-ku, Okayama 700-8558, Japan; Department of Thoracic Surgery, Okayama University Hospital, 2-5-1 Shikata-cho, Kita-ku, Okayama 700-8558, Japan; Department of General Thoracic Surgery and Brest and Endocrinological Surgery, Okayama University Graduate School of Medicine, Dentistry and Pharmaceutical Sciences, 2-5-1 Shikata-cho, Kita-ku, Okayama 700-8558, Japan; Department of Thoracic Surgery, Okayama University Hospital, 2-5-1 Shikata-cho, Kita-ku, Okayama 700-8558, Japan; Department of General Thoracic Surgery and Brest and Endocrinological Surgery, Okayama University Graduate School of Medicine, Dentistry and Pharmaceutical Sciences, 2-5-1 Shikata-cho, Kita-ku, Okayama 700-8558, Japan; Department of Thoracic Surgery, Okayama University Hospital, 2-5-1 Shikata-cho, Kita-ku, Okayama 700-8558, Japan; Department of General Thoracic Surgery and Brest and Endocrinological Surgery, Okayama University Graduate School of Medicine, Dentistry and Pharmaceutical Sciences, 2-5-1 Shikata-cho, Kita-ku, Okayama 700-8558, Japan; Department of Thoracic Surgery, Okayama University Hospital, 2-5-1 Shikata-cho, Kita-ku, Okayama 700-8558, Japan; Department of General Thoracic Surgery and Brest and Endocrinological Surgery, Okayama University Graduate School of Medicine, Dentistry and Pharmaceutical Sciences, 2-5-1 Shikata-cho, Kita-ku, Okayama 700-8558, Japan; Department of Thoracic Surgery, Okayama University Hospital, 2-5-1 Shikata-cho, Kita-ku, Okayama 700-8558, Japan; Department of General Thoracic Surgery and Brest and Endocrinological Surgery, Okayama University Graduate School of Medicine, Dentistry and Pharmaceutical Sciences, 2-5-1 Shikata-cho, Kita-ku, Okayama 700-8558, Japan; Department of Thoracic Surgery, Okayama University Hospital, 2-5-1 Shikata-cho, Kita-ku, Okayama 700-8558, Japan

**Keywords:** neutrophil-to-lymphocyte ratio (NLR), tumor-infiltrating lymphocytes (TILs), non-small cell lung cancer (NSCLC)

## Abstract

**Background:**

Neutrophil-to-lymphocyte ratio (NLR) has been reported as a prognostic biomarker in non-small cell lung cancer (NSCLC); however, the underlying biological rationale remains unclear. The present study aimed to explore the potential utility of NLR as a surrogate biomarker for immune response to cancer and to elucidate the underlying mechanism.

**Methods:**

This retrospective study included the medical records of 120 patients with NSCLC who underwent surgery at the study institution in 2012. NLR in peripheral blood was determined from blood test within 30 days before surgery. Tumor immune status was evaluated using immunohistochemical staining to identify CD3+, CD8+ and FOXP3+ tumor-infiltrating lymphocytes (TILs), and the relationship of NLR, with clinicopathologic characteristics including 5-year overall survival (OS), and the tumor immune status was investigated. The median values of NLR and TIL count were used as cutoff points.

**Results:**

The 5-year OS was significantly better in patients with low NLR (<2.2) than in those with high NLR (≥2.2) (70.1% vs. 56.8%, *P* = 0.042) and in patients with high CD3+ TIL count (≥242) than in those with low CD3+ TIL count (<242) (70% vs. 56.8%, *P* = 0.019). Additionally, the CD3+ TIL count was negatively correlated with preoperative NLR (*P* = 0.005).

**Conclusion:**

NLR might potentially reflect the immune status of tumor microenvironment, explaining its impact on prognosis of patients with NSCLC.

## Teaser

We investigated the relationship between NLR and TIL assessment in NSCLC, and found that NLR potentially reflected immune status in tumor microenvironment.

## Introduction

Lung cancer is one of the most commonly diagnosed cancers worldwide and the leading cause of cancer-related deaths (1,2). Non-small cell lung cancer (NSCLC) accounts for 85% of all lung cancers (3). The development and implementation of variety of treatment options, including surgery, chemotherapy, molecularly-targeted therapy, immune checkpoint blockade therapy and radiotherapy, have led to improved survival in patients with NSCLC (4). Recent advances in therapeutic outcomes have come from a deeper understanding of interactions between cancer cells and the tumor microenvironment (TME), notably immune cells (5,6). Specifically, immune checkpoint inhibitors (ICIs) targeting programmed death receptor, program death ligand-1 (PD-L1) and cytotoxic T-lymphocyte-associated protein 4 receptor are currently used as standard treatment for advanced NSCLC, also being considered for adjuvant and neoadjuvant therapy (7,8). Despite the significant beneficial impact of ICIs, treatment response and overall survival (OS) rates remain unsatisfactory in patients with NSCLC. Studies have proposed the utility of several biomarkers, such as PD-L1 expression in cancer cells and tumor mutation burden for selection of patients who might preferentially benefit from treatment; however, controversies remain (9–11). Therefore, easily measurable predictive biomarkers for response to ICIs is needed to implement precise medical treatment.

Based on its correlation with poor OS, recent studies have proposed neutrophil-to-lymphocyte ratio (NLR), which is calculated by dividing neutrophil count to lymphocyte count in peripheral blood, as a prognostic biomarker in several cancer types, including gastroesophageal, pancreatic, cholangial, hepatocellular, colorectal and renal cell cancers; mesothelioma; and NSCLC (12,13). However, the mechanism underlying the prognostic significance of NLR in OS remains unclear. NLR was initially considered as a parameter that potentially reflected the degree of stress and systemic inflammation in critically ill patients (14). Inflammation is an important factor for tumorigenesis and tumor growth in patients with cancer. Normally, inflammation is essential for tissue repair, regeneration and remodeling, whereas inflammatory responses have powerful mechanisms that lead to the accumulation of mutations and various epigenetic changes in adjacent epithelial cells, resulting in tumorigenesis (15). Furthermore, inflammatory substances also act as tumor growth factors and promote tumor progression and metastasis. Cancer cells interact with surrounding stromal, inflammatory and immune cells to form the TME. Sustained chronic inflammatory response in TME induces immune exhaustion and promotes cancer by blocking anti-tumor immunity, thereby shaping the TME toward a more tumor-permissive state (16–20). Therefore, accumulating data indicate the critical role of inflammation and immune status of the TME for tumor growth and the prognosis in patients with cancer.

Although several studies have examined the relationship between NLR and inflammation, few studies elucidated the association between NLR and immune status in cancer (12,21,22). Here, we investigated the relationship between NLR and tumor-infiltrating lymphocytes (TILs), which are the main players in tumor immunity. Recent studies suggests that T cells especially reflect the immune status in TME and one of the prognostic biomarkers in cancer (23); therefore, we explored the potential utility of NLR as a surrogate biomarker for immune response to cancer with a focus on specific TIL subsets.

## Patients and methods

### Patient selection

This was a retrospective cohort study including 120 patients with NSCLC who underwent surgery at Okayama University Hospital, Okayama, Japan, from 1 January to 31 December 2012. The study was approved by the Ethics Committee of Okayama University Graduate School of Medicine, Dentistry and Pharmaceutical Sciences and Okayama University Hospital, Okayama, Japan (approval no: K2208-072). Oral informed consent was obtained from study participants regarding analyses using surgically resected specimens, due to the absence of invasive procedures needed for specimen collection.

### Date collection

The following clinical data was collected from the medical record: age, sex, performance status (PS) score, smoking history, comorbidities, surgical procedure, histology, pathological stage, adjuvant therapy and NLR. Comorbidity was defined as having one or more of the following: liver dysfunction, dialysis, interstitial pneumonia, ischemic heart disease, malignant disease, cranial nerve disease, cerebrovascular disease, diabetes, anemia, autoimmune disease, arrhythmia and hypertension. Surgical procedure was divided into wadge resection group and segmentectomy or lobectomy group. The International Association of the Study of Lung Cancer TNM staging system for NSCLC (7th edition) was used to determine disease stage (24). NLR was defined as absolute neutrophil count divided by absolute lymphocyte count and determined in peripheral blood samples collected within 30 days before surgery. Patients were categorized into high and low NLR groups, including 61 and 59 patients, respectively, based on a median NLR of 2.2 determined in the present study cohort.

### Immunohistochemistry

Surgical specimens of all study patients were retrospectively evaluated for diagnostic confirmation and immunohistochemical staining. First, formalin-fixed paraffin-embedded tissue specimens were cut into 4-μm-thick sections, mounted on glass slides, and stained with hematoxylin and eosin (HE). The sections were reviewed to confirm histopathological diagnosis and adequacy of specimens for immunohistochemical analysis. Second, for each patient, a representative tissue block containing adequate cancer cells and non-neoplastic lung tissue was selected for immunohistochemical staining. Briefly, formalin-fixed paraffin-embedded sections were deparaffinized with xylene, and rehydrated in graded alcohol series and water. Next, antigen retrieval was performed using a microwave oven for 20 min. Endogenous peroxide activity was blocked by incubating the sections with a 3.0% H_2_O_2_ solution for 5 min. Following a blocking step with 2.5% normal horse serum, the sections were incubated overnight at 4°C with one of the following monoclonal antibodies: anti-CD3ε (D7A6E™; 1:200, Cell Signaling Technology, Danvers, MA, USA), anti-CD8α (C8/144B; 1:200, Cell Signaling Technology) and anti-FOXP3 (236A/E7; 1:200 Abcam, Cambridge, United Kingdom). All antibodies were used at a dilution of 1:200. After twice rinses in phosphate-buffered saline, the sections were incubated with secondary antibody (Vector Laboratories, Burlingame, USA) for 30 min at room temperature. Antibody binding was detected using the ImmPACT DAB peroxidase substrate kit (Vector Laboratories), Following counterstaining using Mayer’s hematoxylin the sections were dehydrated, dried and sealed. Sections incubated without primary antibodies were included as negative control for all evaluated antibodies.

### Evaluation of TILs

International TILs working group had showed how to evaluate TILs with HE stains (25). In the recommendations, only TILs inside the boundaries of the invasive tumor should be evaluated and that Immune infiltrates outside of the tumor borders should be not included. Additional recommendations are exclusion of areas of necrosis or fibrosis from the evaluation. Accordingly, we only assessed lymphocyte infiltrates within the tumor nest and adjacent stroma in immediate contact with the tumor and excluded areas of massive inflammation or necrosis. In each section immunohistochemically stained for CD3, CD8 and FOXP3, the number of TILs was determined by counting positively stained cells in four randomly selected areas using a standard light microscope at 400× magnifications and the average TIL count was determined in each section for further statistical analyses.

### Statistical analyses

All statistical analyses were performed using GraphPad Prism, version 9.4.0 (GraphPad Software, San Diego, CA, USA). OS was defined as the time interval from the date of surgery to the date of death or last follow-up. OS curves were estimated using the Kaplan–Meier method and compared using the log-rank test. The association between clinicopathological characteristics and NLR or TIL count was examine using the Mann–Whitney *U* test. The correlation between NLR and TIL count was examined using Pearson’s correlation coefficient. A *P* value <0.05 was considered to indicate statistical significance.

## Results

### Patient characteristics and NLR

We reviewed the medical records of 120 patients with NSCLC who underwent lung resection. Patient characteristics are summarized in Table 1. Briefly, 76 (63%) patients were male and the median age was 69 (range, 41–87) years. The PS score was 0 in 106 (88%) patients, whereas the PS score range from 1 to 3 in the remaining 14 (12%) patients. Eighty-one (68%) patients were smokers. One hundred and five (88%) patients had some comorbidity. Fifteen patients (12%) underwent wedge resection. A total of 91 (76%) patients were diagnosed with adenocarcinoma. The pathological stage was I in 90 (75%) patients, including 73 (61%) and 17 (14%) patients with stage IA and IB, respectively. Stage IV included five patients with brain or adrenal oligometastasis. Twenty-six patients (22%) received adjuvant therapy. However, information on adjuvant therapy could not be collected for three patients. The median NLR was 2.2 (range, 0.81–5.5). The median observation period was 60.6 (range 0–99.8) months, and recurrence developed in 22 patients. The 5-year OS rate was 63.4%.

**Table 1:** Patient characteristics and univariate analysis for overall survival

Characteristics	*n*	5-year OS (%)	95% CI	*P* value
Sex				0.002
Male	76	53.6	41.5–64.2	
Female	44	80.8	65.2–89.9	
Age (years)				0.02
≤ 69	61	69.5	56.0–79.6	
≥ 70	59	57.0	43.0–68.8	
Performance status				0.23
0	106	65.3	55.1–73.7	
1–3	14	50.0	22.9–72.2	
Smoking history				< 0.001
Smoker	81	52.8	41.2–63.2	
Non smoker	39	85.8	69.0–93.9	
Comorbidities				0.20
Yes	105	60.9	50.5–69.7	
No	15	80.0	50.0–93.1	
Surgical procedure				0.56
Wedge resection	15	65.2	35.1–83.9	
Segmentectomy or lobectomy	105	63.1	52.8–71.7	
Histological subtypes				< 0.001
Adenocarcinoma	91	73.2	62.5–81.3	
Non-adenocarcinoma	29	34.5	18.2–51.4	
Pathological stage (7th)				< 0.001
I	90	75.4	64.8–83.2	
II–IV	30	28.1	13.4–44.9	
Adjuvant therapy				0.39
Yes	26	53.1	31.4–70.8	
No	91	67.2	54.6–76.0	
NLR				0.042
≥ 2.2 (High)	61	56.8	43.1–68.4	
< 2.2 (Low)	59	70.1	56.3–80.3	

^*^Comorbidity was defined as having one or more of the following: liver dysfunction, dialysis, interstitial pneumonia, ischaemic heart disease, malignant disease, cranial nerve disease, cerebrovascular disease, diabetes, anaemia, autoimmune disease, arrhythmia, hypertension.

**Figure 1:**
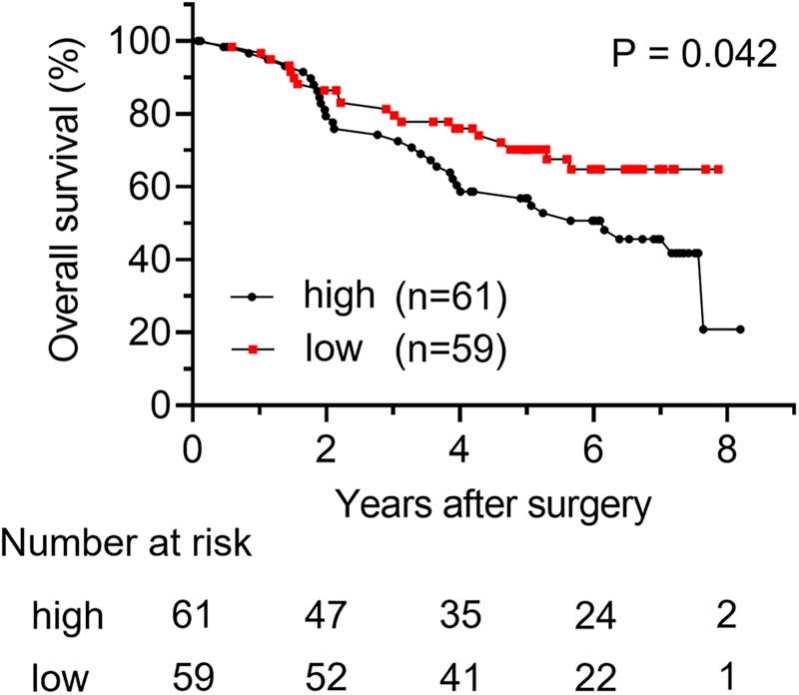
Overall survival (OS) stratified by the value of NLR.

OS curves of the patients categorized according to NLR are shown in Fig. 1. Briefly, the 5-year OS rate was significantly better in patients with low NLR than in those with high NLR (70.1% vs. 56.8%, respectively; *P* = 0.042). Additionally, cancer-specific survival curves of the patients categorized according to NLR are shown in Supplementary Fig. 1(A). There was no significant difference between the two groups. However, the 5-year cancer-specific survival rate tended to be better in patients with low NLR than in those with high NLR (90.3% vs. 80.6%, respectively; *P* = 0.103). Relapse-free survival (RFS) curves of the patients categorized according to NLR are shown in Supplementary Fig. 1(B). There was no significant difference between the two groups. However, 5-year RFS rate tended to be better in patients with low NLR than in those with high NLR (65.3% vs. 53.4%, respectively; *P* = 0.13). As shown in Table 2, analysis of the relationship of NLR with clinicopathological characteristics using the Mann–Whitney *U* test indicated that the rate of patients with higher PS score, non-adenocarcinoma and advanced TNM stage tended to be higher NLR (*P* = 0.014, *P* = 0.019 and *P* < 0.001, respectively).

**Table 2:** The association between characteristics and preoperative NLR

Variables	*n*	NLR		
		Median	Range	*P* value
Sex				0.88
Male	76	2.18	0.88–5.00	
Female	44	2.22	0.81–5.50	
Age (years)				0.43
≤ 69	61	2.26	0.81–5.50	
≥ 70	59	2.06	0.88–4.45	
Performance status				0.014
0	106	2.03	0.81–5.50	
1–3	14	2.77	1.62–4.45	
Smoking history				0.093
Smoker	81	2.85	0.88–5.50	
Non smoker	39	2.00	0.81–5.33	
Comorbidities				0.35
Yes	105	2.23	0.88–5.50	
No	15	1.88	0.81–3.2	
Histological subtypes				0.019
Adenocarcinoma	91	2.09	0.81–5.50	
Non adenocarcinoma	29	2.65	1.40–5.00	
Pathological stage (7th)				< 0.001
I	90	2.00	0.81–5.33	
II–IV	30	2.85	0.88–5.50	

^*^Comorbidity was defined as having one or more of the following: liver dysfunction, dialysis, interstitial pneumonia, ischaemic heart disease, malignant disease, cranial nerve disease, cerebrovascular disease, diabetes, anaemia, autoimmune disease, arrhythmia, hypertension.

### Density of TILs in NSCLC

**Figure 2:**
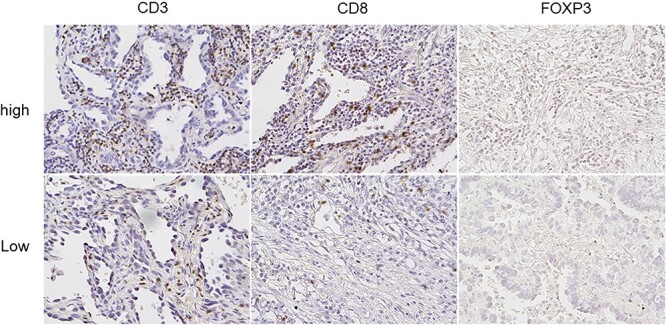
Immunohistochemical stains for TILs (×400).

To determine the mechanism underlying the prognostic significance of NLR in 5-years OS, we next investigated the association of NLR with tumor immune status. To this end, we defined lymphocyte subsets using the NLCLC tissue sections, which were immunohistochemically stained using specific antibodies against CD3, CD8 and FOXP3. CD3 is expressed on nearly all T cells, whereas CD8 and FOXP3 are expressed on cytotoxic and regulatory T cells, respectively. Immune cells infiltrating into tumor tissue displayed broad inter-individual differences in the density of immunohistochemically stained cells, with CD3+, CD8+ and FOXP3+ cell counts varying significantly among the samples. Figure 2 shows representative images of the sections illustrating the distribution of TILs in these tumors. The relationship between the specific TIL counts and the clinicopathological characteristics was analyzed using Mann–Whitney *U* test (Table 3). The median CD3+, CD8+ and FOXP3+ TIL counts were 242 (range, 33–825), 112 (range, 4–550) and 7.5 (range, 0–196), respectively. None of the clinicopathological factors were associated with CD3+ and CD8+ TIL counts, whereas higher FOXP3+ TIL count was significantly associated with male sex (*P* = 0.048), smoking history (*P* = 0.013) and non-adenocarcinoma (*P* = 0.001).

**Table 3:** The association between each characteristic and TIL subset

Variables	*n*	CD3+ TILs			CD8+ TILs			FOXP3+ TILs		
		Median	Range	*P* value	Median	Range	*P* value	Median	Range	*P* value
Sex				0.58			0.55			0.048
Male	76	242	66–825		115	43–450		10	0–196	
Female	44	242	33–547		110	4–352		6	2–69	
Age (years)				0.58			0.54			0.11
≤ 69	61	232	33–825		111	4–415		6	0–196	
≥ 70	59	309	56–790		116	35–550		10	0–116	
Performance status				0.50			0.23			0.61
0	106	245	33–825		116	4–550		8	0–196	
1–3	14	204	56–616		97	49–348		6	0–116	
Smoking history				0.50			0.66			0.013
Smoker	81	229	56–825		110	43–550		10	0–196	
Non smoker	39	283	33–547		136	4–352		5	0–38	
Histological subtypes				0.70			0.74			0.001
Adenocarcinoma	91	245	33–825		116	4–550		6	0–196	
Non adenocarcinoma	29	217	95–601		110	51–381		14	0–116	
Pathological stage (7th)				0.051			0.11			0.055
I	90	281	33–825		119	4–187		7	0–196	
II–IV	30	204	56–601		98	44–415		13	0–116	

### Prognostic value of TILs

**Figure 3:**
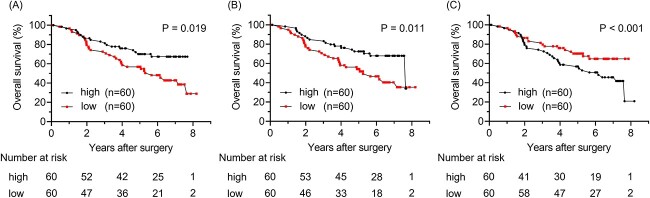
(A) OS stratified by the counts of CD3+ TILs; (B) OS stratified by the counts of CD8+ TILs; (C) OS stratified by the counts of FOXP3+ TILs

OS curve analysis was performed to confirm the prognostic value of each TIL subset in NSCLC. The median cell count was used as the cutoff for each TIL subset. OS curves of the patients categorized according to the number of specific TILs are shown in Fig. 3. The 5-year OS of patients with low and high CD3+ TIL counts were 56.8 and 70%, respectively (*P* = 0.019) (Fig. 3A). The 5-year OS of patients with low and high CD8+ TIL counts were 54.1% and 72.4%, respectively (*P* = 0.011) (Fig. 3B). The 5-year OS of patients with low and high FOXP3+ TIL counts were 79.6 and 46.2%, respectively (*P* = 0.0006) (Fig. 3C). These results indicated that high CD3+, high CD8+ and low FOXP3+ TIL counts were associated with significantly better 5-year OS in the present study cohort. Additionally, RSF curves of the patients categorized according to each TIL subset are shown in Supplementary Fig. 2. The 5-year RFS rate of patients with low and high CD3+ TIL counts were 53.4 and 65.3%, respectively (*P* = 0.094) (Supplementary Fig. 2A). The 5-year RFS rate of patients with low and high CD8+ TIL counts were 47.8 and 70.7%, respectively (*P* = 0.0086) (Supplementary Fig. 2B). The 5-year RFS rate of patients with low and high FOXP3+ TIL counts were 72.8 and 45.5%, respectively (*P* = 0.0013) (Supplementary Fig. 2C).

### Correlation between TILs and NLR

**Figure 4:**
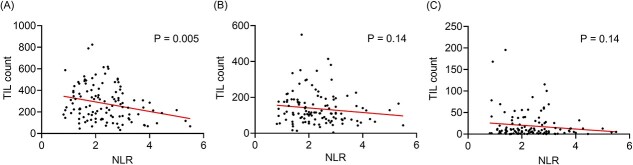
(A) The correlation between the value of NLR and the counts of CD3+ TILs; (B) the correlation between the value of NLR and the counts of CD8+ TILs; (C) the correlation between the value of NLR and the counts of FOXP3+ TILs.

Finally, we examined the relationship between TILs and NLR to assess the potential utility of NLR as a surrogate biomarker for the tumor immune status. The correlation between specific TIL counts and preoperative NLR value was analyzed using Pearson’s correlation coefficient. A lower NLR was correlated with a higher CD3+ TIL count (*P* = 0.005) (Fig. 4A), whereas NLR was not correlated with CD8+ and FOXP3+ TIL counts (Fig. 4B and C). Additionally, according to THE ROC curve analysis, when the cut-off values are set at 2.15 for NLR and 309 for CD3+ TILs, the sensitivity and specificity of the NLR for predicting CD3+ TILs are 63.8 and 60.3%, respectively (AUC = 0.644). These results indicated that NLR partially reflected the anti-tumor immune status, and exhibited potential utility as a convenient test that could be performed using peripheral blood samples to assess immune status of the TME.

## Discussion

Consistent with recent studies reporting the utility of NLR as a prognostic biomarker for various cancer types (12), A high NLR was also associated with poor prognosis in NSCLC in the current study. NLR is considered as one of the peripheral biomarkers for cancer-related inflammation, including lymphocyte-to-monocyte ratio, platelet-to-lymphocyte ratio and Glasgow Prognostic Score (26–29). However, the mechanism underlying the association of high NLR with poor outcomes in cancer remains unclear.

NLR reflects the quantitative relationship between neutrophils and lymphocytes in peripheral blood. Neutrophils are considered the main immune cells related to inflammation that protects the body from microbial infection and eliminates pathogens. In addition, many studies have shown that not only macrophages but also neutrophils can reshape their own phenotype and function during the interaction that take place between cancer cells and the TME. These studies demonstrate that neutrophils can participate in tumor development through a variety of mechanisms, including THE promotion of proliferation, migration and invasion of cancer cells and formation of new blood vessels in tumor (30–32). Lymphocytes play a key role in the protective immunity by inhibiting the proliferation and migration of cancer cells. Specifically, T cells can produce cytotoxic factors and cytokines that eliminate cancer cells. However, failure of cancer cell elimination by the immune system lead to T cell exhaustion and changes in some T cell subsets which suppress immune response to cancer cells, resulting in a reduction in the number of TILs (16–20). Based on these findings, NLR is a potential biomarker reflecting the immune status of TME, related to prognosis in patients with cancer.

We performed immunohistochemical staining for CD3, CD8 and FOXP3 in TILs to evaluate the immune status of TME in patient with NSCLC. CD3 is expressed on almost all T cells, CD8 is expressed on cytotoxic T cells, which can potentially kill cancer cells, and FOXP3 is expressed on regulatory T cells, which downregulateS immune response (33,34). Several reports have shown that these TIL subsets reflect the immune status of TME and are associated with prognosis in several cancer types, including blest and colorectal cancers, mesothelioma and NSCLC. Specific TIL subsets that have been shown to be associated with cancer prognosis vary across the studies and include CD3+, CD4+, CD5+, CD8+, CD45RA+, FOXP3+ and PD1+ TILs; however, CD3+, CD8+ and FOXP3+ TILs were commonly shown to associate with prognosis in many cancer types (23,35–38). In agreement with previous reports, we also found that low CD3+ and CD8+ TIL density, and high FOXP3+ TIL density were associated with poor prognosis in NSCLC.

We investigated the relationship of NLR with these TIL subsets to explore its potential utility as a surrogate biomarker for immune response to cancer. Our analysis indicated that a significant negative correlation between NLR and CD3+ TIL count. On the other hand, we did not find a correlation between NLR and the number of CD8+ or FOXP3+ TILs. It is satisfied that NLR evaluated in peripheral blood can’t perfectly reflect the immune status of TME. A previous study reported that the distribution of specific subsets of T cells, including CD4+, CD8+ and FOXP3+ T cells, was different between peripheral blood and the TME (39). However, the negative correlation between NLR and CD3+ TIL count detected in the present study suggests that we can roughly presume the density of T cells in TME from the value of NLR in peripheral blood. Therefore, it is possible that NLR might partially reflect the anti-tumor immunity as a potential mechanism underlying its association with prognosis in cancer. As reported in previous studies, the density of CD8^+^ or FOXP3^+^ TILs can sharply reflect the immune status of TME and prognosis in patients with cancer (23,36). However, determining the density of CD8^+^ or FOXP3^+^ TILs in clinical settings requires the evaluation of tumor tissue with substantial time and effort and is not cost-effective, whereas peripheral blood NLR can be easily determined using preoperative laboratory data without the need for tumor tissue. Furthermore, our findings that NLR can partially reflect anti-tumor immunity is likely to be useful in considering treatment options, including ICIs, for patients with cancer (10,33,40).

Most of the several studies investigating the relationship between NLR and TILs used semi-quantitative evaluation of TIL density determined with HE staining, and few studies determined TIL density using immunohistochemical staining (41,42). Dirican et al. reported the negative correlation between intra-tumor CD3+ TILs and NLR in untreated patients with advanced-stage NSCLC (43); however, the cohort size was small and the TIL counts were evaluated in biopsy specimens of patients with advanced cancer. Gawinski et al. showed the negative correlation between a level of CD3+ lymphocytes in the center of tumors and pre-treatment NLR in patients with left-side colorectal cancer, but no correlation between CD8+ lymphocytes with NLR (44). In another study, high CD3+ TIL count was significantly correlated with low NLR in locally advanced triple-negative breast cancer (45). However, consistent with the current study results, the authors failed to demonstrate a relationship of NLR with CD8+ and FOXP3+ TIL counts.

Our study has several limitations. First, this was a retrospective study based on a database of a modest sample size from a single institution. In RFS analyses, THE 5-year RFS rate tended to be better in patients with low NLR or with high CD3+ TIL, although there was no significant difference. This result may be due to the inconsistent timing of periodic postoperative examinations in this study cohort. We acknowledged that this is a limitation of retrospective studies. Second, the definition and the methods used to evaluate TILs vary among studies. Although we evaluated TILs by counting the number of immunoreactive lymphocytes in four randomly selected areas in each section, this approach might have introduced a certain degree of allocation bias due to tumor heterogeneity. Finally, there are currently no established cutoff values for NLR and TILs. Therefore, these results should be repeated in larger cohorts with established settings. However, it is worth nothing that we performed a novel study on the subject that is a few in the literature. We explored the rationale for peripheral blood NLR as a surrogate biomarker for prognosis based on its association with CD3+, CD8+ and FOXP3+ TILs in resected NSCLC tissue specimens.

In conclusion, the present study not only revealed that low NLR and high CD3+ TIL density was both associated with good prognosis but also found that CD3+ TIL count was negatively correlated with the value of NLR in patients with NSCLC. These findings suggest that the value of NLR can partially reflect the immune status of TME, explaining its impact on prognosis of patients with NSCLC. In addition, although further investigation is needed, we can roughly presume immune status of TME in patients with cancer from the value of NLR, and that may be useful in management of treatment options.

## Supplementary Material

Supplementary_Figure_1_hyae058

Supplementary_Figure_2_hyae058
