# Supplementary figures and images for: Utility of neutrophil-to-lymphocyte ratio as an indicator of tumor immune status in non-small cell lung cancer
Source: Jpn J Clin Oncol. 2024 May 4;54(8):895–902. doi: 10.1093/jjco/hyae058 (PMC11322889; doi:10.1093/jjco/hyae058)

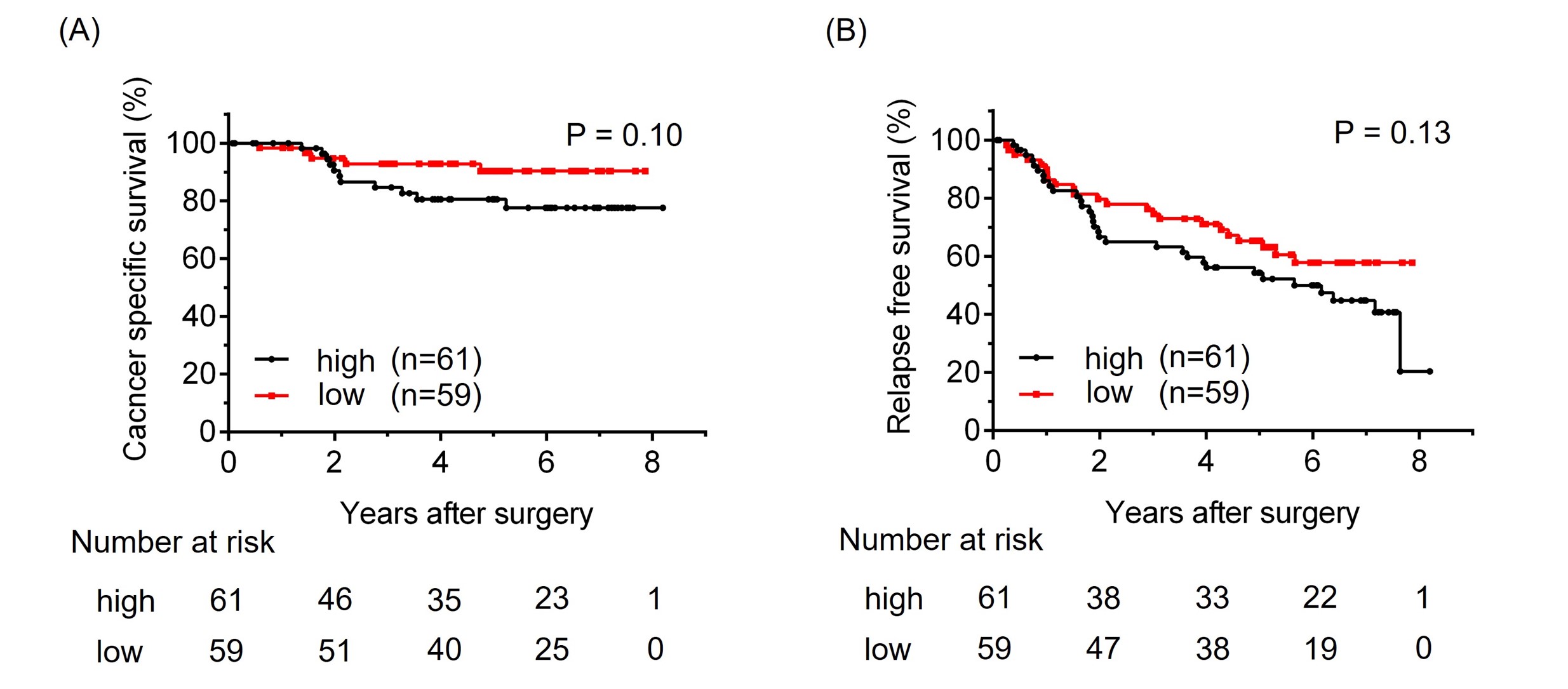

Supplement: Supplementary_Figure_1_hyae058 [file supplementary_figure_1_hyae058.jpeg]

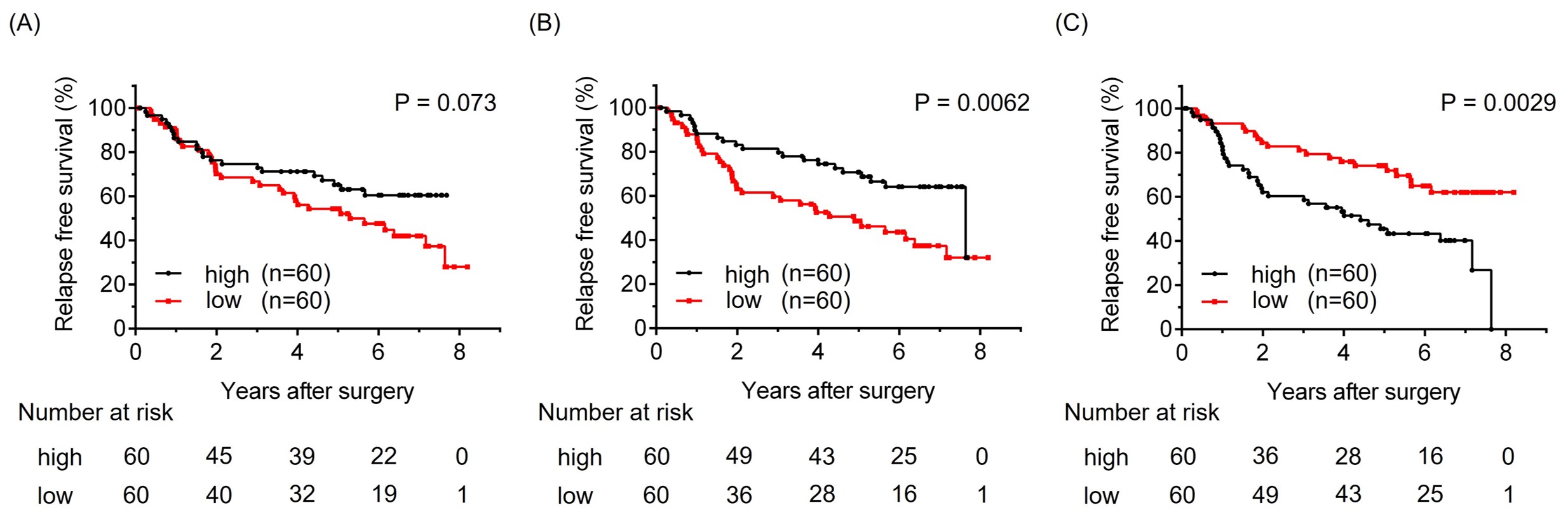

Supplement: Supplementary_Figure_2_hyae058 [file supplementary_figure_2_hyae058.jpeg]
